# Sports participation and myocarditis: Influence of sport types on disease severity

**DOI:** 10.1016/j.ijcha.2021.100895

**Published:** 2021-10-26

**Authors:** Robin Bouchau, Eve Cariou, Antoine Deney, Slimane Belaid, Romain Itier, Virginie Blanchard, Pauline Fournier, Alexandre Duparc, Michel Galinier, Didier Carrié, Olivier Lairez, Yoan Lavie-Badie

**Affiliations:** a Department of Cardiology, Rangueil University Hospital, Toulouse, France; b Department of Nuclear Medicine, Rangueil University Hospital, Toulouse, France; c Medical School of Toulouse, Paul Sabatier University, Toulouse, France; d Department of Cardiovascular Rehabilitation, Rangueil University Hospital, Toulouse, France

**Keywords:** Sport, Myocarditis, Mitchell’s Classification, AM, Acute myocarditis, CMR, Cardiac magnetic resonance, IQR, Interquartile Range, LGE, late gadolinium enhancement, LVEF, Left ventricular ejection fraction

## Abstract

**Objective:**

To study, in the context of acute myocarditis (AM) in sportsmen, the association between the category of sport practiced and the severity of AM.

**Design:**

Retrospective study.

**Setting:**

Single tertiary center.

**Patients:**

82 sportspeople (≥2.5 h of sport per week) who presented an AM.

**Assessment of Risk Factors:**

The type of sport activity before AM were collected.

**Main Outcome Measures:**

Complicated AM was defined by a left ventricular ejection fraction <50% or the use of inotropic drugs or ventricular assist device.

**Results:**

Mean age was 29 ± 9 year-old, 77 (94%) were men. Sixteen (20%) had a complicated AM. Practicing sport over 6 h a week or a Mitchell’s Class IIIA sport were associated with complicated AM (44 vs. 17%, p = 0.019 and 38 vs. 11%, p = 0.008, respectively). Practicing a Mitchell’s Class IC sport was associated with uncomplicated AM (6 vs. 38%, p = 0.008).

**Conclusion:**

In sportspeople's AM, the pattern of sport activity (static or dynamic component, practice intensity) is associated with the disease’s severity.

## 1 Introduction

Acute myocarditis (AM) is an inflammatory disease of the myocardium, which is commonly related to viral infections or immune-mediated diseases [1], [2]. It mainly affects young males [3] and its incidence is 22 per 100,000 people i.e. approximately [4]. AM in athletes is a source of concern, from diagnosis to sport return and medical follow up [5], [6], [7]. Postmortem studies in athletes who experienced sudden death, shown that AM was diagnoses up for 7–10% of cases [8], [9], [10], [11]. Moreover, intense endurance training may increase the susceptibility to viral infections [12]. The links between sports practice and AM are both close and poorly understood.

The purpose of this study was to evaluate, in sportsmen who experienced AM, the relationship between the category of sport practiced and the severity of the disease.

## 2 Methods

### 2.1 Population

We retrospectively analyzed all hospital records from consecutive adult patients, hospitalized in the Toulouse University Hospital, from 1st January 2009 to 31st December 2019, with an International Classification of Diseases 10th revision (ICD10) separation diagnosis of AM (I40.0, I41.1, I40.8 or I41.8).

To retain the diagnosis of AM and be included in the study, patients had to have the following criteria:

– Participation in sports ≥ 2.5 h per week

And

– The onset of symptoms < 1 month from hospitalization.
– A positive endomyocardial biopsy (according to the Dallas criteria [13]) or a positive cardiac magnetic resonance (CMR) (2 or 3 Lake Louise Criteria [14]) combined with an elevation of blood troponin (Ic or Tc) above the 99th percentile.

To avoid any possible confusion with differential diagnostics, we excluded patients over 70 years old, patients with significant coronary artery disease, patients over 50 year-old without coronary imaging and patients with alternative diagnosis (e.g stress cardiomyopathy).

### 2.2 Data collection

All subjects were contacted by phone calls and submitted a standardized questionary regarding their sport activity at the time of diagnosis of AM. Data relative to medical history and AM were collected via the electronic medical records software and hospital database.

The study is conformed to the principles outlined in the Declaration of Helsinki. It is registered in the register of retrospective studies of the Toulouse University Hospital (number’s register: RnIPH 2020-125) and cover by the MR-004 (French National Commission for Informatics and Liberties number: 2206723 v 0). This study was approved by Toulouse University Hospital and confirms that ethic requirements were totally respected in this report.

### 2.3 Physical and sports activity quantification

Participation in sport was defined by practicing sports for an average ≥2.5 h per week during the previous six months at the time of diagnosis of AM.

Patients were then divided, as suggested by McKinney et al. [15], in “exercisers” (≥2.5 h/week of physical activity with the primary aim to maintain health and fitness), “recreational athletes” (≥4 h/week for pleasure, fitness, or unregulated competitions) and “competitive athletes” (≥6 h/week and participation to official competitions).

Participation in sport was classified according to Mitchell’s classification [16], in 9 groups, differentiated with their static (I, II, III) and dynamic component (A, B, C):

– I: Low < 20% of maximum voluntary contraction,
– II: moderate = 20–50% of maximum voluntary contraction,
– III: High > 50% of maximum voluntary contraction
– A: low < 40% of maximum O_2_ uptake,
– B: moderate = 40–70% of maximum O_2_ uptake,
– C: High > 70% of maximum O_2_ uptake.

We also collected the intensity of training (hours per week), competition participation, doping attitudes. Finally, follow-up data, recovery of left ventricular function and AM relapses, as well as a possible return to sport were collected.

### 2.4 Endpoints

The primary endpoint was related to the acute phase of AM and was defined as the occurrence of complicated AM. Complicated AM was defined as one of the following criteria: left ventricular ejection fraction (LVEF) < 50%, use of dobutamine or noradrenaline intravenous drug, or use of cardiac assistance device (heart pump percutaneous support system – Impella® – or extracorporeal membrane oxygenation).

### 2.5 Statistical analysis

Continuous variables were expressed as means ± standard deviation or as medians with interquartile ranges (IQR) when not normally distributed. Nominal variables were expressed as numbers and percentages. The Association between the mean values of continuous variables was assessed using the Mann-Whitney rank sum test. Nominal variables were investigated by the χ^2^ test or the Fisher exact test when appropriate. The association between intensity (>6 h) and type (Mitchell class III sport, that accounts for most of individuals with complicated AM, versus other disciplines) was tested with a multivariate analyse using logistic regression. P-value inferior to 0.05 was considered significant. The software XLSTATS v2019.1 (Addinsoft, Paris, FR) was used for statistical analysis.

## 3 Results

### 3.1 Population characteristics

**Fig. 1:**
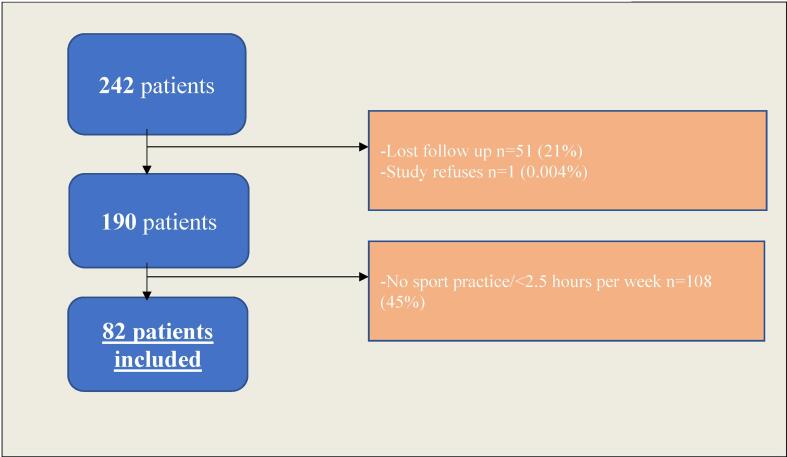
Flow diagram illustrating screening and inclusion criteria.

Two hundred forty-two patients presented an AM, without exclusion criteria, over the study period. Among them, 51 (21%) were lost to follow-up and 1 refused to participate to the study. Finally, among the 190 remaining patients, 82 (43%) met the definition of sportspeople and were finally included. The flowchart of the study is presented in Fig. 1.

### 3.2 Baseline characteristics

Participants were predominantly young (29 ± 9-year-old) males (n = 77, 94%). Tobacco was the main cardiovascular risk factor (34%), followed by family coronary artery disease history (7%). Most of the patients had electrocardiogram abnormalities (67%), mainly regarding ST segment (58%) and T wave (14%). Two (2%) patients have autoimmune disorders (1 Ankylosing spondylitis and 1 scleroderma).

All population baseline characteristics are depicted in Table 1.

**Table 1:** Characteristics.

	TOTAL POPULATION	COMPLICATED MYOCARDITIS	UNCOMPLICATED MYOCARDITIS	*p* value
	n = 82	n = 16	n = 66	
**Baseline**				
Age at diagnosis, years	29.1 ± 9.4	29.8 ± 11.02	28.9 ± 9	*0.870*
Male, n (%)	77 (94)	15 (93)	62 (94)	*0.977*
Body mass index, kg/m^2^	24.9 ± 3.6	24 ± 2.2	25.1 ± 3.9	*0.323*

**Cardiovascular history, n (%)**				
Tobacco	29 (35)	7 (44)	22 (33)	*0.434*
Cannabis	3 (4)	1 (6)	2 (3)	*0.538*
Dyslipidémia,	3 (4)	1 (6)	2 (3)	*0.538*
Hypertension	1 (1)	1 (6)	0	***0.035***
Coronary heart disease family history	6 (7)	1 (6)	5 (8)	*0.855*
Diabetes mellitus	0	0	0	
History of myocarditis	3 (4)	1 (6)	2 (3)	*0.538*
Systemic disease	2 (2)	1 (6)	1 (1)	*0.271*
Immunodepression	2 (2)	0	2 (3)	*0.481*

**Laboratory testing**				
Troponine T peak, ng/L	1015 [510–1483]	580 [403.5–1094]	1040 [580–1529.5]	*0.298*
C reactive protein peak, mg/L	38.5 [12.3–99]	91 [40–197]	29 [9–73]	***0.013***

**Electrocardiogram**				
Abnormal electrocardiogram	55 (67)	12 (75)	43 (65)	*0.266*
Left bundel branch block	1 (1)	1 (6)	0	***0.035***
Right bundel branch block	5 (6)	0	5 (8)	*0.271*
T wave abnormability	12 (15)	1 (6)	11 (17)	*0.325*
ST abnormability	48 (58)	9 (56)	39 (59)	*0.948*
Ventricular arrhythmias	2 (2)	1 (6)	1 (1)	*0.246*
Atrioventricular block	1 (1)	1 (6)	0	***0.035***

**Echocardiographic imaging**				
LVEF %	54.4 ± 10.9	41 ± 9	57.6 ± 6.6	***<0.0001***
Pericardial effusion, n (%)	3 (4)	0	3 (5)	*0.381*
Wall motion abnormality, n (%)	16 (19)	5 (31)	11 (17)	*0.197*

**Cardiac magnetic resonance**				
Hyperemia, n (%)	43 (52)	8 (50)	35 (53)	*0.971*
Edema, n (%)	71 (87)	15 (94)	56 (85)	*0.796*
Subepicardial LGE, n (%)	79 [96]	15 (94)	64 (97)	*0.538*
Mid wall LGE, n (%)	14 (17)	6 (37)	8 (12)	***0.016***
Subendocardial LGE, n (%)	2 (2)	1 (6)	1 (1)	*0.271*
Septal LGE, n (%)	12 (15)	7 (44)	5 (8)	***<0.001***
Inferior LGE, n (%)	57 (69)	11 (69)	46 (70)	*0.941*
Lateral LGE, n (%)	70 (85)	12 (75)	58 (88)	*0.191*
Anterior LGE, n (%)	18 (22)	4 (25)	14 (21)	*0.743*
Apical LGE, n (%)	51 (62)	10 (62)	41 (62)	*0.978*
LVEF %	54.8 ± 8.6	42.8 ± 8.7	57.8 ± 5.4	***<0.0001***
LVDVi, ml/m^2^	86.9 ± 15.4	93 ± 20	85.4 ± 13.9	*0.294*
Average segments affected in LGE	5 [2–7]	5 [3–8.5]	5 [2.3–6]	*0.368*

**Medical therapy after hospitalization**				
ACE Inhibitor, n (%)	77 (94)	14 (87)	63 (95)	*0.233*
ACE Inhibitor average duration – months	6 [4–9]	6 [6–8]	6 [4–9.8]	*0.720*
Beta-blocker, n (%)	73 (89)	13 (81.3)	60 (91)	*0.267*
Beta-blocker average duration – months	6 [4–10]	8 [6–12]	6 [4–10]	*0.291*

Values are mean ± SD, n (%), or median [interquartile range]. Values in bold are significant.

LVEF = Left Ventricular Ejection Fraction; LGE = Late Gadolinium enhancement; LVEDVi = indexed Left Ventricular Diastolic Volume; ACE = Angiotensin-Converting Enzyme.

### 3.3 Sport characteristics

Before AM, the median time of sport participation was 5.4 ± 3.7 h/week. Twenty-nine (35%) were “exercicers”, 39 (47%) “recreational athletes”, and 14 (17%) “competitive athletes”. Mitchell’s class IC (n = 26, 32%) was the most represented category with the following main sports: soccer (n = 15, 18%) and running (n = 10, 12%). The second most practiced class was IIB (n = 14, 17%) represented by rugby (n = 10, 12%) and fitness (n = 3, 4%). Finally, Mitchell's class IIIA included 13 (16%) patients, mainly represented by weightlifting (n = 10, 12%).

**Fig. 2:**
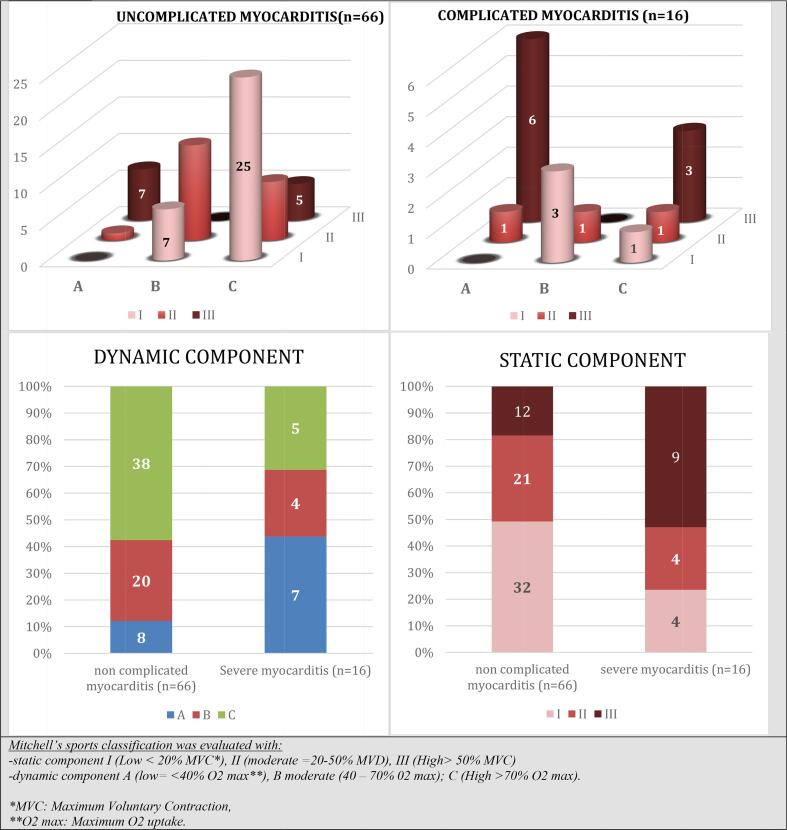
Baseline sports classification in study population according to am Severity.

All other sports characteristics are resumes in Table 2 and Fig. 2.

**Table 2:** Relationship between the type of sport before AM and complicated AM.

		Whole POPULATION	COMPLICATED acute MYOCARDITIS	NON COMPLICATED acute MYOCARDITIS	*p* value
		n = 16	n = 82	n = 66	
Time of practice – hours		5.4 ± 3.7	6.4 ± 4.1	5.1 ± 3.6	*0.269*
Practice >6 h a week		18 (22)	7 (44)	11 (17)	***0.019***
Competition		26 (32)	5 (31)	21 (32)	*0.965*
Use doping substance		3 (4)	1 (6)	2 (3)	*0.538*
Exercisers		29 (35)	5 (31)	24 (36)	*0.701*
Recreational athletes		39 (47)	8 (50)	31 (47)	*0.828*
Competitive athletes		14 (17)	3 (19)	11 (17)	*0.843*

Mitchell’s classification	IA	0	0	0	
	IB	10 (12)	3 (19)	7 (11)	*0.372*
	IC	26 (32)	1 (6)	25 (38)	***0.015***
	IIA	2 (2)	1 (6)	1 (1)	*0.271*
	IIB	14 (17)	1 (6)	13 (20)	*0.200*
	IIC	9 (11)	1 (6)	8 (12)	*0.500*
	IIIA	13 (16)	6 (37)	7 (11)	***0.008***
	IIIB	0	0	0	
	IIIC	8 (10)	3 (19)	5 (8)	*0.177*

*Mitchell’s sports classification was evaluated with:*					
– static component I (Low < 20% MVC*), II (moderate = 20–50% MVD), III (High > 50% MVC)					
– dynamic component A (low = <40% VO2 max**), B moderate (40–70 % V02 max); C (High > 70% VO2 max)					

* MVC: Maximum Voluntary Contraction.

** VO2 max: Maximum O2 uptake.

### 3.4 Primary endpoint

Sixteen (20%) patients presented a complicated AM. Sport participation patterns before AM were significantly associated with complicated AM. Indeed, practicing sport over 6 h by week was associated with complicated AM (44 vs. 17%, P = 0.019), as well as the practice of Mitchell’s class IIIA sport (6 (37%) vs. 7 (11%) p = 0.008). Contrariwise, Mitchell’s class IC sports were associated with non-complicated AM (1 (6%) vs 25 (38%) p = 0.015).

After multivariate analysis, Michell class 3 sport participation remained associated with complicated AM (Odds ratio 5.2 (95 %CI 1.5–17.3), p < 0.01) in contrast to practice intensity >6 h (Odds ratio 3.3 (95 %CI 0.9–11.7), p = 0.06).

### 3.5 Follow-up

The median follow-up was 2.8 years (IQR: 1.6–5). No deaths occurred during the period. Seven (8%) patients experienced recurrence of AM. Sixty-seven (81%) patients had a follow-up CMR at 6 months from AM and 48 (71%) among them had persistent LGE. Only 1(1%) patient did not normalize his LVEF. Sixty-five (79%) sportspeople returned to sport after healing, after a median period of sport’s restriction of 6 months (IQR: 3.8–6.8). Thirty-five (43%) return to sport at the same level as before AM. Eighteen (22%) return to competitive activity. There was no impact of the return to sport on the recurrence of AM (6 (9%) vs 3 (18%), p 0.37).

## 4 Discussion

In this retrospective study that included 82 sportspeople who experienced an AM, we described interactions between the way of practicing sports and AM severity. Main results can be summarized as follows: (1) Practice of high intensity power sports (Mitchell’s class III sports) was associated with complicated AM. (2) Practice of high intensity endurance sports (Mitchell’s class IC sports) was associated with uncomplicated AM. (3) A high practice frequency (>6 h/week) trended to be associated with complicated AM

Complicated forms of myocarditis are usually associated with more extensive myocardial lesions [17]. Such lesions can be attributable to an overlap between pathogens (mainly viral replication) and immunity (cell injuries induced by natural killer cells, macrophages, and activate virus-specific T cells) [1], [18]. Regarding links between pathogens and sport, we can hypothesize that sport promotes the cellular penetration of the virus. Indeed, murine models showed higher viral titers and larger myocardial fibrosis in exercised mice infected by coxsackievirus virus [19]. Secondly, participation in sport affects immune system modulation [20]. For example, practicing a high intensity endurance sport can result in a significant lymphocytopenia (decrease in circulating T cells and natural killer cells), which persists throughout the 3.5 h of recovery [21]. Experimentally, in exercise-aggravated coxsackievirus B3 murine myocarditis, T lymphocyte suppression can lead to a decrease of myocardial inflammation and necrosis [22]. Therefore, high intensity endurance sports may be protective against complicated forms of AM. On the other hand, practicing a high intensity power sport leads to lymphocytosis (mainly natural killer cells) followed by a rapid return to normal (30 min) [23]. The promotion of natural killer cells, involved in immune-induced lesions in myocarditis, could explain the association between severe forms and power sports.

AM are mainly viral but can be related to toxics. The use of doping substances can have harmful effects on the heart [24]. In our study, very few patients admitted to consuming such substances. However, it is likely that this use was hidden. The practice of high intensity power sports (e.g. weight lifting) and a high practice frequency are potentially associated with doping [25], [26]. It is therefore possible that this is an explanatory factor to our observations.

Finally, it is now recognized that certain cardiomyopathies, in particular arrhythmogenic cardiopathies, first evolve as inflammatory episodes [27]. It is admitted that high intensity physical exercise has an aggravative effect in these diseases [28], [29]. It is therefore possible that some AM labeled as complicated are in fact such cardiomyopathies, aggravated by intense sport practice.

## 5 Limitations

Two main limitations are the small sample size and the retrospective nature of the study. Indeed, the retrospective nature of this registry may have introduced potential bias, for example, data about sport practice could have been distorted by a lack of memories for patients who had AM longer ago. Sportspeople population selection inherently leads to a potential selection bias. However, baseline characteristics between sports and non-sports people were similar (Supplemental Data File).

## 6 Conclusion

In the setting of sportspeople AM, the pattern of sport participation is associated with the presentation’s severity.

## Disclosure of funding

This research received no specific grant from public, commercial, or non-profit funding agencies.

## Declaration of Competing Interest

The authors report no relationships that could be construed as a conflict of interest.

## Appendix A Supplementary material

Supplementary data 1 Characteristics of athletes vs non-athletes (excluded).
