# Supplementary material for: Sports participation and myocarditis: Influence of sport types on disease severity
Source: Int J Cardiol Heart Vasc. 2021 Oct 26;37:100895. doi: 10.1016/j.ijcha.2021.100895 (PMC8571161; doi:10.1016/j.ijcha.2021.100895)
Supplement: Supplementary data 1 — Characteristics of athletes vs non-athletes (excluded). [file mmc1.docx]

Appendix: Characteristics of athletes vs non-athletes(excluded)

|  | **TOTAL**  **n=190** | **Sportspeople**  **n=82** | **Non-sportspeople**  **n=108** | ***p value*** |
| --- | --- | --- | --- | --- |
| Baseline |  |  |  |  |
| Age at diagnosis,- years | 30 ± 10.4 | 29.1 ± 9.3 | 30.7 ± 11.1 | *0.309* |
| Male | 159 (83.7) | 77 (94) | 82 (76) | ***0.001*** |
| Body mass index - kg/m2 | 24.7 ± 3.9 | 24.9 ± 3.6 | 24.5 ± 4.2 |  |
| Cardiovascular history |  |  |  |  |
| Tobacco | 79 (41.5) | 29 (35.4) | 50 (46.3) | *0.287* |
| Cannabis | 13 (6.8) | 3 (3.7) | 10 (9.6) | *0.130* |
| Dyslipidémia, | 10 (5.3) | 3 (3.7) | 7 (6.5) | *0.388* |
| Hypertension | 5 (2.6) | 1 (1.2) | 4 (3.7) | *0.289* |
| Coronary heart disease family history | 15 (7.9) | 6 (7.3) | 9 (8.3) | *0.797* |
| Diabetes mellitus | 2 (1.1) | 0 | 2 (1.9) | *0.215* |
| History of myocarditis | 8 (4.2) | 3 (3.7) | 5 (4.63) | *0.741* |
| Systemic disease | 10 (5.3) | 2 (2.4) | 8 (7.4) | *0.129* |
| Immunodepression | 3 (1.6) | 2 (2.4) | 1 (0.9) | *0.407* |
| *Laboratory testing* | | | | |
| Troponine T peak – ng/L | 920 [384 – 1594] | 1015 [510 - 1483] | 644 [282.8 - 1599] | *0.317* |
| C reactive protein peak – mg/L | 32 [13 - 86] | 38.5 [12.3 - 99] | 61.7 [12.6 – 68.8] | *0.465* |
| Electrocardiogram |  |  |  |  |
| Abnormal electrocardiogram | 123 (6.5) | 55 (67.1) | 68 (63) | *0.592* |
| Left bundel branch block | 1 (1.1) | 1 (1.2) | 0 | *0.251* |
| Right bundel branch block | 14 (7.4) | 5 (6.1) | 9 (8.3) | *0.551* |
| T wave abnormability | 26 (13.7) | 12 (14.63) | 14 (13) | *0.753* |
| ST abnormability | 104 (54.7) | 48 (58.4) | 56 (51.9) | *0.454* |
| Ventricular arrhythmias | 3 (1.6) | 2 (2.4) | 1 (0.9) | *0.411* |
| Atrioventricular block | 3 (1.6) | 1 (1.2) | 2 (1.9) | *0.725* |
| *Echocardiographic imaging* | | | | |
| LVEF % | 55 ± 10.9 | 54.4 ± 10.9 | 55.5 ± 10.4 | *0.281* |
| Pericardial effusion | 7 (3 .6) | 3 (3.7) | 4 (3.7) | *0.957* |
| Kinetic trouble | 42 (22.1) | 16 (19.5) | 26 (27.1) | *0.317* |
| Magnetic résonnance Imaging (MRI) |  |  |  |  |
| Hyperemia | 110 (57.9) | 43 (52.4) | 67 (62) | *0.137* |
| Edema | 156 (82.1) | 71 (86.6) | 85 (78.7) | *0.247* |
| Subepicardial LGE | 184 (96.9) | 79 [96.3] | 105 (97.2) | *0.201* |
| Midwall LGE, | 36 (19.4) | 14 (17.1) | 22 (20.4) | *0.525* |
| Subendocardial LGE | 11 (5.8) | 2 (2.4) | 9 (8.3) | *0.080* |
| Septal LGE | 29 (15.3) | 12 (14.6) | 17 (15.7) | *0.690* |
| Inferior LGE | 118 (62.1) | 57 (69.5) | 61 (56.5) | *0.079* |
| Lateral LGE | 162 (85.2) | 70 (85.4) | 92 (85) | *0.905* |
| Anterior LGE | 52 (27.3) | 18 (21.6) | 34 (31.5) | *0.134* |
| Apical LGE | 120 (63.1) | 51 (62.2) | 69 (63.9) | *0.746* |
| SQS score / 68 | 12.1 ± 8,6 | 10,6 ± 6.6 | 13,22 ± 9.6 | *0.114* |
| SQS % | 17 ± 12.4 | 15.6 ± 9.7 | 19.4 ± 14.1 | *0.114* |
| LVEF % | 54.6 ± 8.9 | 54.8 ± 8.6 | 54.46 ± 9.2 | *0.938* |
| LVEF < 40% | 11 (5.8) | 5 (6.1) | 6 (5.5) | *0.877* |
| Average segments affected in LGE | 5 [3 - 8] | 5 [2 - 7] | 5.5 [3 – 8] | *0.150* |
| Medical therapy during hospitalisation |  |  |  |  |
| Inotropic agents | 9 (4.7) | 5 (6.1) | 4 (3.7) | *0.442* |
| ECMO | 6 (3.2) | 3 (3.6) | 3 (2.8) | *0.731* |
| Medical therapy after hospitalisation |  |  |  |  |
| ACE Inhibitor | 181 (95.3) | 77 (93.9) | 104 (96.3) | 0.442 |
| ACE Inhibitor average duration - months | 6 [4 - 11] | 6 [4 - 9] | 6 [5 - 12] | 0.287 |
| Beta blocker | 171 (90) | 73 (89) | 98 (90,7) | 0.696 |
| Beta blocker average duration - months | 6 [4 – 11.5] | 6 [4 - 10] | 6 [6 - 12] | 0.356 |
| Calcium channel blocker | 6 (3.2) | 2 (2.4) | 4 (3,7) | 0.831 |
| Calcium channel average duration - months | 6 [4.5 – 11.5] | 15.5 [13.3 – 17.8] | 6 [3 - 6] | 0.095 |
| *Values are mean ± SD, n (%), or median [interquartile range]. Values in* ***bold*** *are significant* | | | | |
